# Uric acid-based nutritional and inflammatory biomarkers for predicting cardiovascular-kidney-metabolic syndrome and mortality: a population-based study with external validation

**DOI:** 10.3389/fendo.2026.1937815

**Published:** 2026-09-22

**Authors:** Yi Deng, Miao Hu, Tianran Ren, Lin Qian, Yingying Xie, Song Wen, Ling Zhou

**Affiliations:** 1 Department of Nephrology, The First Affiliated Hospital of Soochow University, Suzhou, China; 2 Department of Cardiology, Fuwai Hospital, National Center for Cardiovascular Diseases, Chinese Academy of Medical Sciences and Peking Union Medical College, Beijing, China

**Keywords:** cardiovascular-kidney-metabolic syndrome (CKM), Inflammation, NHANES, nutrition, uric acid-to-albumin ratio (UAR), Uric acid-to-high-density lipoprotein cholesterol ratio (UHR)

## Abstract

**Background:**

Uric acid-to-HDL cholesterol ratio (UHR) and uric acid-to-albumin ratio (UAR) are emerging composite biomarkers of nutrition and metabolic inflammation. This study investigates and compares their associations with advanced cardiovascular-kidney-metabolic (CKM) syndrome and all-cause mortality.

**Methods:**

This cross-sectional study included 17,833 adults from NHANES 2001-2020, with external validation in a clinical cohort of 300 hospitalized patients. Weighted logistic regression, restricted cubic spline (RCS), ROC curve, and Cox regression analyses were performed.

**Results:**

Both UHR and UAR were significantly elevated in patients with advanced CKM syndrome (both P < 0.001) and independently associated with increased CKM risk (UHR: OR = 1.07, 95%CI: 1.06-1.09; UAR: OR = 1.08, 95%CI: 1.06-1.10). Nonlinear associations were observed for both biomarkers, with inflection points at 9.46 for UHR and 15.79 for UAR (both P for nonlinear < 0.001). In ROC analysis, UAR slightly outperformed UHR in the NHANES cohort (AUC: 0.640 vs. 0.602), whereas UHR showed superior performance in the clinical cohort (AUC: 0.787 vs. 0.686). Both biomarkers remained independently associated with all-cause mortality after full adjustment.

**Conclusion:**

UHR and UAR are independently associated with advanced CKM syndrome and have complementary clinical utility. UAR may be preferred for early screening, whereas UHR may be better suited for CKM diagnosis and metabolic risk stratification. Their combined use may facilitate prognosis evaluation and mortality risk warning.

## 1 Introduction

Cardiovascular-kidney-metabolic (CKM) syndrome, as an emerging clinical concept, summarizes the complex pathophysiological phenomena among obesity, type 2 diabetes (T2DM), chronic kidney disease (CKD) and cardiovascular disease (CVD). CKM syndrome is not only a major contributor to the global burden of disease, but also represents a vicious cycle among metabolic disorders, chronic inflammation and organ damage (1). From 2011 to 2020, nearly 90% of the population met the diagnostic criteria for stage 1 or higher of CKM syndrome. Simultaneously, 14.6% of U.S. adults were classified as having late-stage CKM syndrome (stage 3 or 4) (2). Therefore, identifying simple, cost-effective, and reproducible biomarkers to facilitate early detection and risk stratification of CKM syndrome is imperative.

Uric acid (UA) and high-density lipoprotein cholesterol (HDL-C) are two routinely measured biomarkers in clinical practice. UA, the end product of purine metabolism, has been implicated in oxidative stress, endothelial dysfunction, and pro-inflammatory responses, all of which are key drivers of CKM pathophysiology (3–5). Conversely, HDL-C exerts anti-inflammatory, antioxidant, and anti-atherogenic effects, and low levels of HDL-C are associated with adverse cardiometabolic outcomes (6–8). The ratio of uric acid to high-density lipoprotein cholesterol (UHR) has recently emerged as a novel integrated biomarker that combines the detrimental effects of elevated UA with the protective deficiency of low HDL-C, potentially offering a more comprehensive reflection of metabolic and inflammatory status than either indicator alone (9, 10).

Unlike HDL-C, which primarily serves as a marker of lipid-related anti-inflammatory capacity, serum albumin (ALB) is another easily accessible clinical parameter. ALB not only exhibits potent antioxidant and anti-inflammatory properties but also reflects nutritional status, thereby exerting protective effects in cardiovascular and kidney diseases (11, 12). The uric acid-to-albumin ratio (UAR), serving as another novel composite biomarker in the present study, captures the integrated status of nutrition, inflammation, and metabolism (13).

To date, numerous studies have demonstrated that UHR and UAR are closely associated with CVD, CKD, and metabolic disorders. Fan et al. reported that UHR was an independent risk factor for CKD (14). In addition, UHR was shown to predict CVD events and death in patients with CKD (15). Moreover, it has also been shown to be associated with the risk of CVD and dyslipidemia, the risk of myocardial infarction in patients with diabetes, and the risk of peripheral arterial disease in patients with hypertension (16–18). Similarly, studies have shown that UAR levels are positively correlated with the severity of peripheral arterial disease and coronary artery disease, and are significantly associated with carotid intima-media thickness (19–21). Moreover, UAR can be used to predict all-cause and cardiovascular mortality in U.S. adults (22). Despite the established associations of UHR and UAR with the aforementioned diseases, no study has specifically investigated their association with CKM syndrome.

Therefore, the present study aimed to investigate the independent associations of UHR and UAR with advanced CKM syndrome using data from the National Health and Nutrition Examination Survey (NHANES) 2001-2020, and to explore the potential nonlinear dose-response relationships as well as the predictive performance of UHR and UAR for advanced CKM syndrome. Additionally, we sought to examine their associations with all-cause mortality. Furthermore, we attempted to validate the above findings in an independent clinical cohort of hospitalized patients from the Departments of Cardiology, Nephrology, and Endocrinology at The First Affiliated Hospital of Soochow University. We hypothesized that elevated UHR and UAR would be independently associated with an increased risk of advanced CKM syndrome as well as an increased risk of all-cause mortality.

## 2 Materials and methods

### 2.1 Study population

The data used in this study were derived from NHANES, conducted by the National Center for Health Statistics (NCHS). NHANES is a comprehensive survey designed to collect nationally representative data on the health and nutritional status of the civilian non-institutionalized U.S. population, including demographics, socioeconomic status, dietary habits, and health-related indicators. We analyzed data from adult participants across the NHANES cycles from 2001 to 2020. The initial sample consisted of 97,657 individuals. Participants younger than 20 years of age (n=4,3793), those with missing data related to CKM syndrome (n=29,109), those with missing data on UA, HDL-C and ALB (n=1,525), and those with missing data on key covariates (n=5,397) were excluded. A final total of 17,833 participants were included in the analysis. A flowchart of the participants selection process is presented in **Figure 1**.

**Figure 1:**
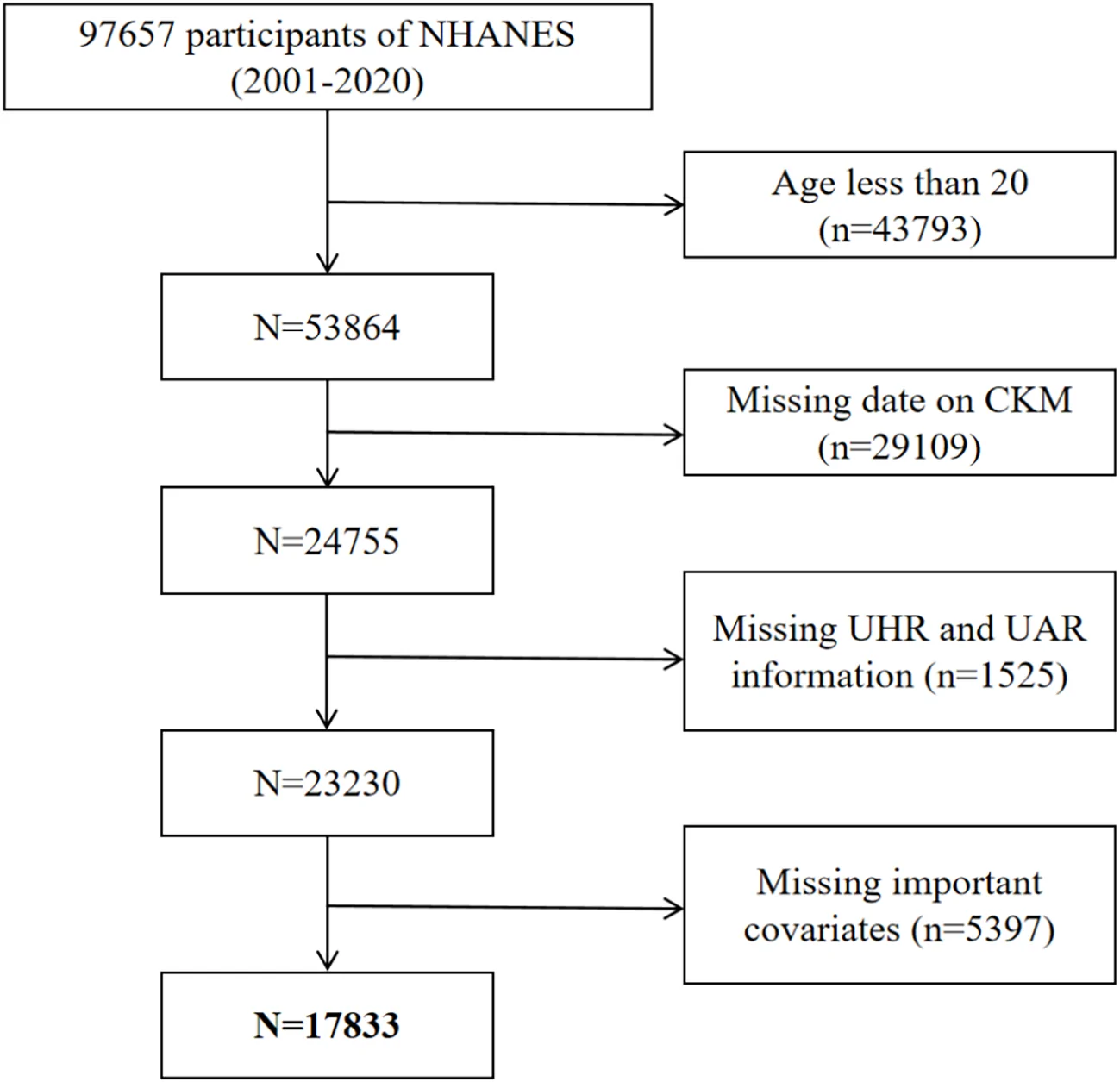
Flow chart of the participants selection process.

To clinically validate the results, we also randomly collected the relevant indicators of inpatients from January to March 2026 at the First Affiliated Hospital of Soochow University, including 100 from the Department of Cardiology, 100 from the Department of Nephrology, and 100 from the Department of Endocrinology. Eligible patients were identified through daily screening of admission records. Inclusion criteria were age ≥ 20 years and complete laboratory and clinical data for UHR, UAR, and CKM staging. Exclusion criteria included acute infection, malignancy, severe liver disease, chronic diarrhea or inflammatory bowel disease, nephrotic syndrome, maintenance dialysis, pregnancy/lactation, and use of immunomodulatory agents within 3 months.

### 2.2 Definition and staging of CKM syndrome

According to the Presidential Advisory published by the American Heart Association (AHA) in 2023, CKM syndrome is classified into five stages (**Supplementary Table S1**). Stage 0 denotes the absence of CKM risk factors, whereas Stage 1 is defined solely by the presence of excess or dysfunctional adiposity. Stage 2 begins to incorporate CKD as a consideration and adopts a more stringent definition of metabolic risk factors. Metabolic risk factors included: (1) hypertriglyceridemia (triglycerides ≥ 135 mg/dL); (2) hypertension (systolic blood pressure ≥ 130 mmHg, diastolic blood pressure ≥ 80 mmHg, or use of antihypertensive medications); (3) diabetes (HbA1c ≥ 6.5%, use of glucose-lowering medication, or self-reported physician diagnosis); and (4) metabolic syndrome. Metabolic syndrome was defined according to the harmonized criteria as the presence of at least three of the following: elevated waist circumference(WC) (≥ 88 cm in women or ≥ 102 cm in men), hypertriglyceridemia (≥ 150 mg/dL), low HDL cholesterol (< 50 mg/dL in women or < 40 mg/dL in men), elevated blood pressure, and elevated fasting glucose (≥ 100 mg/dL) or use of glucose-lowering medications or prior diagnosis of diabetes. Stage 3 is characterized by the presence of subclinical CVD, with a predicted probability of a cardiovascular event exceeding 20% within 10 years using the PREVENT equations, or by stage 4 or 5 CKD. Stage 4 is defined as established clinical CVD in individuals with metabolic risk factors or CKD. including coronary artery disease, heart failure, myocardial infarction, angina pectoris, and stroke. Stage 4 was further subdivided into 4a (without kidney failure; eGFR ≥ 15mL/min/1.73m^2^) and 4b (with kidney failure; eGFR < 15 mL/min/1.73m^2^). In this study, Stages 3 and 4 were combined into advanced CKM syndrome, while Stages 0 through 2 were combined into non-advanced CKM syndrome (1, 23, 24). The detailed criteria for all CKM syndrome stages are provided in **Supplementary Table S1**.

### 2.3 Exposure factors measurement

This study calculated UHR and UAR as exposure variables. All of these indicators were derived from the NHANES laboratory dataset. UA, HDL-C and ALB were measured from fasting blood samples collected in the morning. HDL-C was determined using either the direct enzymatic method or the precipitation method, while UA was measured using the time-point colorimetric method, and ALB was measured using the bromcresol purple method. For the clinical data, we collected UA, HDL-C and ALB values from fasting morning biochemical panel tests of a total of 300 hospitalized patients. The UHR was calculated using the formula: UHR = [UA (mg/dL)/HDL-C (mg/dL)] × 100. The UAR was calculated using the formula: UAR = [UA (mg/dL)/ALB (g/L)] × 100.

### 2.4 Covariates definition

To ensure the robustness of the results, the present study adjusted for a number of covariates, including: age (years), gender, race (Mexican American, other Hispanic, non-Hispanic white, non-Hispanic black, other race), marriage (married, widowed, divorced, separated, never married, living with partner), education (less than 9^th^ grade, 9-11^th^ grade, high school grade/GED or equivalent, some college or AA degree, college graduate or above), poverty-to-income ratio (PIR), smoke, alcohol, body mass index (BMI), and WC. Laboratory measurements included low-density lipoprotein cholesterol (LDL-C), total cholesterol (TC), triglycerides (TG), serum creatinine (SCR), blood urea nitrogen (BUN), glycated hemoglobin A1c (HbA1c), fasting blood glucose (FBG), alanine aminotransferase (ALT), aspartate aminotransferase (AST).

### 2.5 Statistic analysis

All statistical analyses accounted for the complex sampling design of NHANES, and weighted methods were applied to ensure representativeness and robustness. The sampling weights, stratification, and primary sampling units (WTMEC2YR, SDMVSTRA, SDMVPSU) recommended by NCHS were used to reflect the complexity of the survey design. All statistical studies employed the R software (version 4.5.1).

For categorical variables, baseline characteristics were expressed as counts (percentages) and for continuous variables as means (standard deviations). Weighted multivariable logistic regression analysis was used to examine the association between UHR/UAR and advanced CKM syndrome. Model 1: unadjusted; Model 2: adjusted for demographic factors (age, gender, race, marriage, education, and PIR); Model 3: further adjusted for lifestyle (smoke, alcohol). Multicollinearity was checked using variance inflation factors (VIFs), with a threshold of VIF < 5 indicating no significant collinearity. To explore the potential non-linear dose-response relationship between UHR/UAR and the risk of advanced CKM syndrome, the restricted cubic spline (RCS) model with three knots positioned at the 10th, 50th, 90th percentiles of the exposure distribution was used. Subgroup analyses were conducted based on age, gender, race, marriage, education, PIR, smoke and alcohol. To evaluate the association between UHR/UAR and all-cause mortality, Cox proportional hazards regression models were constructed, with the same three adjustment models (Model 1-3) applied. The proportional hazards assumption was tested using Schoenfeld residuals. Survival curves were generated using the Kaplan-Meier method and compared using the log-rank test. Additionally, receiver operating characteristic (ROC) curve analysis was performed to evaluate the predictive value of UHR/UAR for advanced CKM syndrome. Statistical significance was defined as p < 0.05.

## 3 Results

### 3.1 Baseline characteristics of study participants

This study included a total of 17,833 participants from the NHANES database spanning 2001 to 2020, including 3,248 patients with advanced CKM syndrome and 14,585 without advanced CKM syndrome. **Table 1** shows that the mean age of the study population was 47.45± 16.58 years, with 49% male. Demographically, patients with advanced CKM syndrome tended to be older, predominantly male, with a higher proportion of non-Hispanic White individuals, and exhibited higher rates of being widowed, as well as lower education levels and lower PIR. In terms of lifestyle, the proportions of smoking or alcohol consumption were higher. Moreover, these patients had higher BMI and WC, as well as elevated levels of TG, UA, SCR, BUN, FBG, HbA1c and AST, while HDL-C, LDL-C, TC, ALB and ALT levels were lower. Additionally, both UHR and UAR were significantly higher in patients with advanced CKM syndrome (p < 0.001).

**Table 1:** Baseline characteristics of study participants.

Characteristic	Overall (N = 17833)	Without advanced CKM syndrome (N = 14585)	With advanced CKM syndrome (N = 3248)	P-value
Age (years)	47.45± (16.58)	44.56± (15.01)	67.19± (12.96)	<0.001
Gender, n(%)				<0.001
Male	8,940 (49%)	7,026 (48%)	1,914 (56%)	
Female	8,893 (51%)	7,559 (52%)	1,334 (44%)	
Race, n(%)				<0.001
Mexican American	2,811 (7.7%)	2,467 (8.3%)	344 (4.0%)	
Other Hispanic	1,462 (4.8%)	1,251 (5.0%)	211 (3.4%)	
Non-Hispanic white	8,306 (71%)	6,448 (70%)	1,858 (76%)	
Non-Hispanic black	3,591 (10.0%)	2,927 (9.9%)	664 (11%)	
Other race	1,663 (6.4%)	1,492 (6.5%)	171 (5.4%)	
Marriage, n(%)				<0.001
Married	9,750 (58%)	7,958 (58%)	1,792 (60%)	
Widowed	1,882 (7.1%)	1,053 (5.1%)	829 (21%)	
Divorced	2,123 (11%)	1,779 (11%)	344 (9.9%)	
Separated	459 (1.9%)	386 (1.9%)	73 (1.7%)	
Never married	2,495 (15%)	2,364 (16%)	131 (4.0%)	
Living with partner	1,124 (6.8%)	1,045 (7.3%)	79 (3.3%)	
Education, n(%)				<0.001
Less than 9^th^ grade	1,768 (5.1%)	1,241 (4.4%)	527 (9.9%)	
9-11^th^ grade	2,455 (10%)	1,912 (9.7%)	543 (15%)	
High school grade/GED or equivalent	4,144 (24%)	3,307 (23%)	837 (29%)	
Some college or AA degree	5,292 (31%)	4,473 (32%)	819 (27%)	
College graduate or above	4,174 (30%)	3,652 (31%)	522 (20%)	
PIR	3.06± (1.62)	3.12± (1.63)	2.66± (1.54)	<0.001
Alcohol, n(%)				<0.001
Yes	13,414 (79%)	11,101 (80%)	2,313 (72%)	
No	4,419 (21%)	3,484 (20%)	935 (28%)	
Smoke, n(%)				<0.001
Yes	8,291 (47%)	6,348 (45%)	1,943 (62%)	
No	9,542 (53%)	8,237 (55%)	1,305 (38%)	
Hypertension, n(%)				<0.001
Yes	6,475 (32%)	4,255 (27%)	2,220 (67%)	
No	11,358 (68%)	10,330 (73%)	1,028 (33%)	
DM, n(%)				<0.001
Yes	2,174 (8.6%)	1,203 (6.1%)	971 (26%)	
No	15,659 (91%)	13,382 (94%)	2,277 (74%)	
MetS, n(%)				<0.001
Yes	6,427 (33%)	4,492 (29%)	1,935 (60%)	
No	11,406 (67%)	10,093 (71%)	1,313 (40%)	
BMI (kg/m2)	28.84± (6.71)	28.71± (6.69)	29.70± (6.76)	<0.001
WC (cm)	98.86± (16.39)	97.98± (16.27)	104.83± (15.95)	<0.001
HDL-C (mg/dL)	54.24± (16.04)	54.56± (16.06)	52.01± (15.76)	<0.001
LDL-C (mg/dL)	115.06± (35.24)	116.53± (34.66)	105.06± (37.44)	<0.001
TC (mg/dL)	193.36± (40.17)	194.71± (39.56)	184.16± (42.94)	<0.001
TG (mg/dL)	120.30± (67.11)	118.09± (66.34)	135.42± (70.30)	<0.001
UA (mg/dL)	5.48± (1.37)	5.41± (1.33)	5.92± (1.52)	<0.001
SCR (mg/dL)	0.89± (0.35)	0.85± (0.18)	1.11± (0.81)	<0.001
BUN (mg/dL)	13.43± (5.21)	12.83± (4.18)	17.50± (8.63)	<0.001
HbA1c (%)	5.58± (0.88)	5.51± (0.81)	6.07± (1.16)	<0.001
FBG (mmol/L)	5.51± (1.57)	5.39± (1.42)	6.33± (2.20)	<0.001
ALB (g/L)	42.39± (3.23)	42.56± (3.19)	41.26± (3.32)	<0.001
ALT (U/L)	25.36± (25.52)	25.47± (19.37)	24.65± (50.34)	<0.001
AST (U/L)	24.96± (17.16)	24.87± (17.05)	25.55± (17.88)	<0.001
UHR	11.16 ± (4.83)	10.95 ± (4.70)	12.55 ± (5.42)	<0.001
UAR	12.97 ± (3.35)	12.76 ± (3.19)	14.44 ± (3.97)	<0.001

PIR, poverty-to-income ratio; DM, diabetes mellitus; MetS, metabolic syndrome; BMI, body mass index; WC, waist circumference; HDL-C, high-density lipoprotein cholesterol; LDL-C, low-density lipoprotein cholesterol; TC, total cholesterol; TG, triglycerides; UA, serum uric acid; SCR, serum creatinine; BUN, blood urea nitrogen; HbA1c, glycated hemoglobin A1c; FBG, fasting blood glucose; ALB, albumin; ALT, alanine aminotransferase; AST, aspartate aminotransferase; UHR, serum uric acid to high-density lipoprotein cholesterol ratio; UAR, serum uric acid-to-albumin ratio.

### 3.2 Association between UHR, UAR and advanced CKM syndrome

To evaluate the association between UHR, UAR and advanced CKM syndrome, three multivariate logistic regression models were constructed (**Table 2**). In continuous analysis, UHR was independently associated with an increased risk of advanced CKM syndrome in Model 1 (OR = 1.06, 95%CI: 1.05-1.07, P<0.001), Model 2 (OR = 1.07, 95%CI: 1.06-1.09, P<0.001) and fully adjusted Model 3 (OR = 1.07, 95%CI: 1.06-1.09, P<0.001). Quartile-based analysis showed a graded positive association, with the highest quartile of UHR conferring the greatest risk (OR = 2.56, 95%CI: 2.09-3.14, P<0.001), and a significant linear trend (p for trend<0.001`). Similarly, UAR in continuous form was associated with higher risk of advanced CKM syndrome in the three models (all p <0.001). In quartile analysis, only the top quartile of UAR remained significant in Model 3 (OR = 1.78, 95%CI: 1.47-2.16, P<0.001), with a significant linear trend (p for trend<0.001).

**Table 2:** Associations between UHR, UAR and advanced CKM syndrome.

	Model 1		Model 2		Model 3	
	OR (95% CI)	P-value	OR (95% CI)	P-value	OR (95% CI)	P-value
UHR	1.06 (1.05, 1.07)	<0.001	1.07 (1.06, 1.09)	<0.001	1.07 (1.06, 1.09)	<0.001
UHR (quartile)						
Quartile 1	Reference		Reference		Reference	
Quartile 2	1.54 (1.31, 1.81)	<0.001	1.61 (1.29, 2.00)	<0.001	1.60 (1.29, 1.99)	<0.001
Quartile 3	1.83 (1.55, 2.16)	<0.001	1.87 (1.50, 2.35)	<0.001	1.85 (1.48, 2.32)	<0.001
Quartile 4	2.26 (1.94, 2.63)	<0.001	2.55 (2.08, 3.13)	<0.001	2.56 (2.09, 3.14)	<0.001
p for trend		<0.001		<0.001		<0.001
UAR	1.15 (1.13, 1.17)	<0.001	1.08 (1.06, 1.10)	<0.001	1.08 (1.06, 1.10)	<0.001
UAR (quartile)						
Quartile 1	Reference		Reference		Reference	
Quartile 2	1.47 (1.23, 1.76)	<0.001	1.18 (0.94, 1.46)	0.150	1.19 (0.96, 1.48)	0.120
Quartile 3	1.70 (1.44, 2.01)	<0.001	1.09 (0.89, 1.34)	0.400	1.11 (0.91, 1.35)	0.300
Quartile 4	3.10 (2.65, 3.63)	<0.001	1.77 (1.45, 2.15)	<0.001	1.78 (1.47, 2.16)	<0.001
p for trend		<0.001		<0.001		<0.001

Model 1: unadjusted.

Model 2: adjusted for age, gender, race, marriage, education, and PIR.

Model 3: adjusted for age, gender, race, marriage, education, PIR, smoke and alcohol.

### 3.3 Restrained cubic spline regression analysis

Our study further performed RCS analysis to explore the potential nonlinear association between UHR, UAR and the risk of advanced CKM syndrome. Following full adjustment for potential confounders, **Figure 2** showed significant nonlinear dose-response relationships for both UHR and UAR with advanced CKM syndrome (both P for nonlinear < 0.001), with inflection points identified at 9.46 and 15.79, respectively.

**Figure 2:**
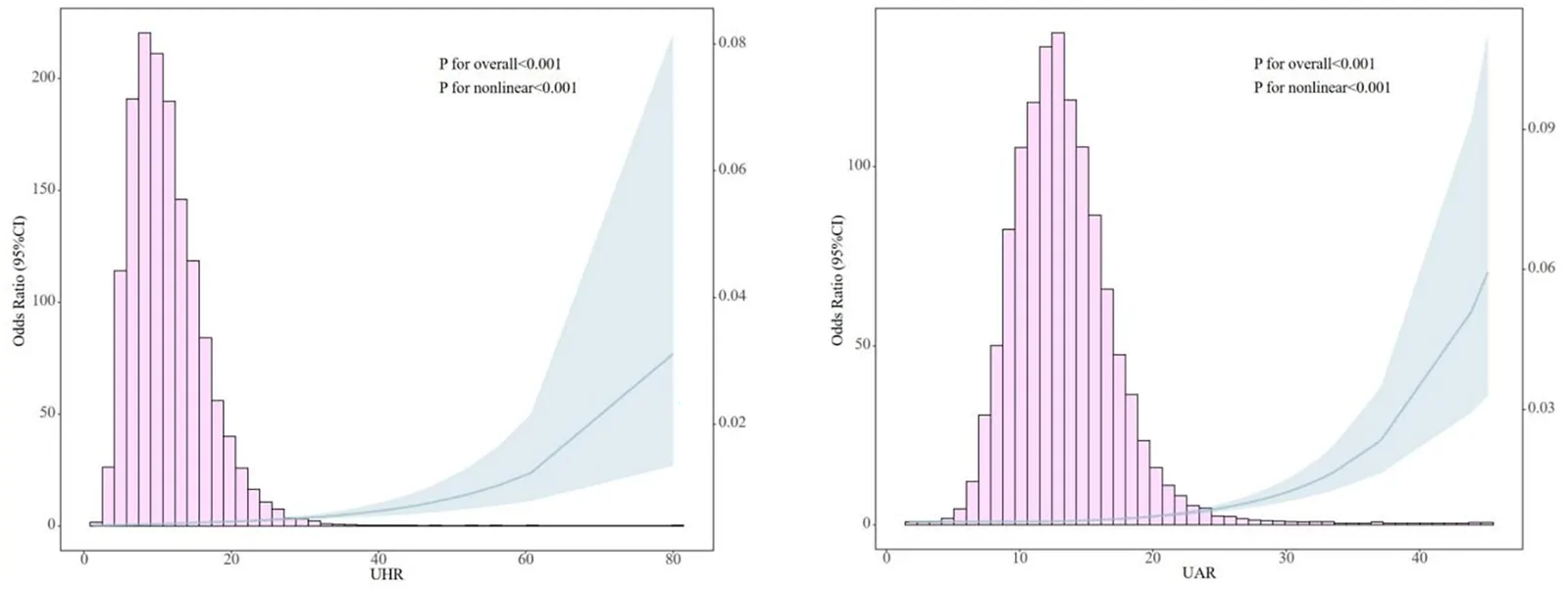
RCS analysis with multivariate-adjusted associations between UHR, UAR and advanced CKM syndrome.

### 3.4 Subgroup analysis

To further evaluate the robustness of the association between UHR, UAR and advanced CKM syndrome and to explore potential effect modifiers, subgroup analyses stratified by age, gender, race, marriage, education, PIR, alcohol and smoke were conducted (**Figure 3**). Significant effect modification was observed for UHR across age, gender, and marriage, and for UAR across race and education. No significant interactions were detected for the remaining subgroups for either biomarker (all P for interaction > 0.05).

**Figure 3:**
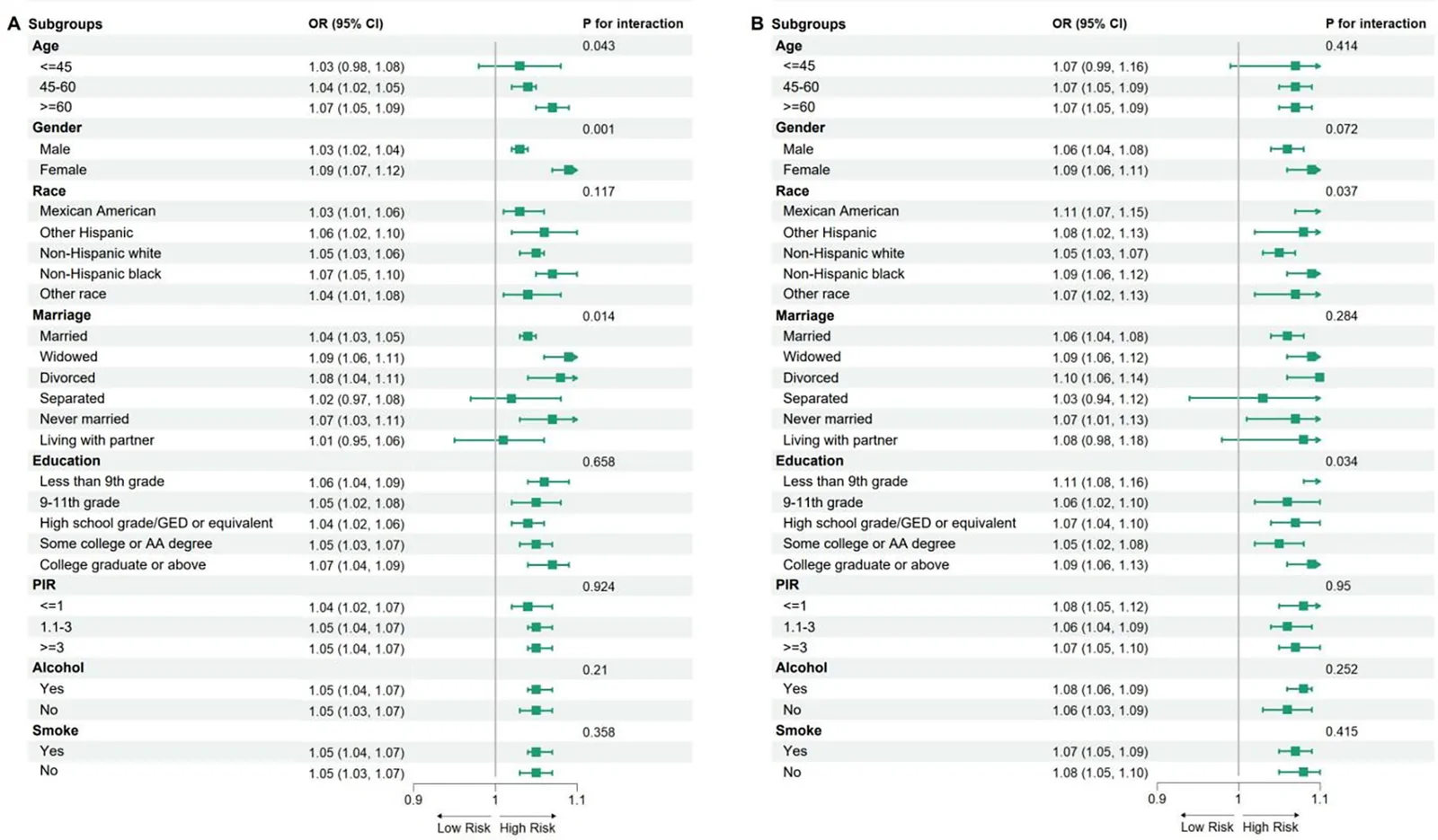
Subgroup analysis for the association between UHR **(A)**, UAR **(B)** and advanced CKM syndrome.

### 3.5 Predictive value of UHR and UAR for advanced CKM syndrome

ROC curve analysis was performed to evaluate the predictive value of UHR and UAR for advanced CKM syndrome. As shown in **Figure 4**, UAR demonstrated a higher area under the curve (AUC) of 0.640 (95% CI: 0.629-0.650) compared to UHR, which had an AUC of 0.602 (95% CI: 0.591-0.612). (p < 0.001) The combination of UHR and UAR yielded an AUC of 0.640 (95% CI: 0.629-0.651), which was nearly identical to that of UAR alone. (p = 0.292).

**Figure 4:**
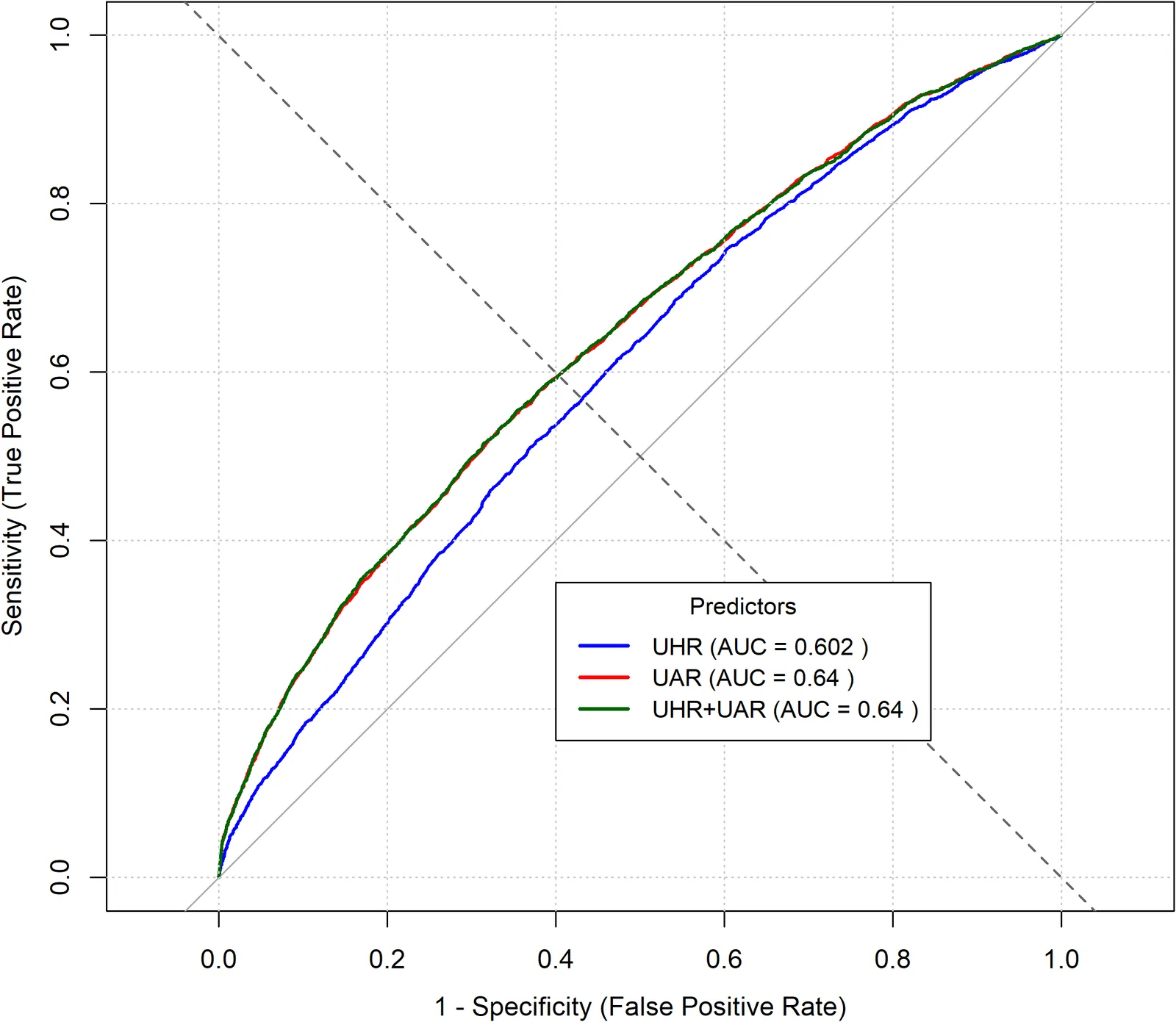
ROC curves of UHR, UAR, and their combination for predicting advanced CKM syndrome.

### 3.6 Association between UHR, UAR and all‐cause death in participants with CKM syndrome

Multivariable Cox regression analyses were performed to examine the independent associations of UHR and UAR with all-cause mortality, with three sequentially adjusted models (**Table 3**). Both UHR and UAR were significantly associated with increased risk of all-cause mortality in participants with CKM syndrome across all three models (all P < 0.001). In the fully adjusted Model 3, per-standard-deviation (per-SD) increase in UHR was associated with a 12% higher risk of all-cause mortality (HR = 1.12, 95% CI: 1.07-1.17). UAR demonstrated a stronger association, with a per-SD increase associated with a 17% higher risk (HR = 1.17, 95% CI: 1.13-1.22).

**Table 3:** Associations between UHR, UAR and all‐cause death in participants with CKM syndrome.

	Model 1		Model 2		Model 3	
	HR (95% CI)	P-value	HR (95% CI)	P-value	HR (95% CI)	P-value
UHR (per-SD)	1.19 (1.15, 1.24)	<0.001	1.12 (1.07, 1.17)	<0.001	1.12 (1.07, 1.17)	<0.001
UAR (per-SD)	1.47 (1.42,1.53)	<0.001	1.18 (1.13, 1.23)	<0.001	1.17 (1.13, 1.22)	<0.001

Model 1: unadjusted.

Model 2: adjusted for age, gender, race, marriage, education, and PIR.

Model 3: adjusted for age, gender, race, marriage, education, PIR, smoke and alcohol.

Kaplan-Meier survival analysis is also employed to evaluate the associations of UHR and UAR with all-cause mortality among participants with CKM syndrome. As shown in **Figure 5**, participants with CKM syndrome were stratified into high and low groups based on the median values of UHR and UAR. The Kaplan-Meier curves demonstrated that individuals with higher UHR levels had significantly lower survival probabilities compared to those with lower UHR levels (log-rank P < 0.0001). Similarly, participants with higher UAR levels exhibited significantly worse survival than those with lower UAR levels (log-rank P < 0.0001). These findings suggest that elevated UHR and UAR are both associated with increased mortality risk in the CKM syndrome population.

**Figure 5:**
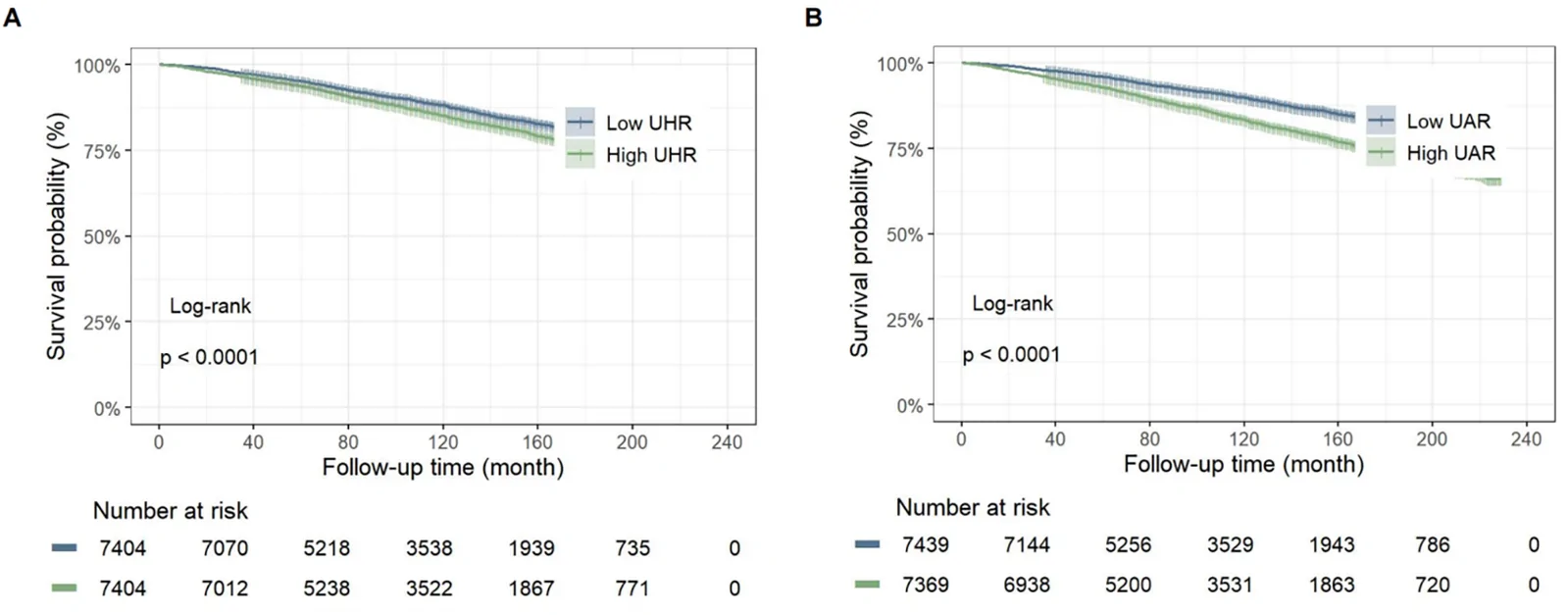
Kaplan-Meier curves of the survival rate with high and low UHR **(A)** and UAR **(B)** values.

### 3.7 Clinical cohort analysis

The clinical cohort included 300 hospitalized patients (96 without advanced CKM syndrome, 204 with advanced CKM syndrome). Compared to patients without advanced CKM syndrome, those with advanced CKM syndrome were significantly older, more likely to be male and smokers, and had a higher prevalence of hypertension but a lower prevalence of diabetes. Regarding laboratory parameters, patients with advanced CKM syndrome had lower levels of HDL-C, LDL-C, and TC, and significantly higher levels of UA, SCR, and BUN (all p < 0.001). Most importantly, both UHR and UAR were significantly elevated in patients with advanced CKM syndrome (**Table 4**).

**Table 4:** Baseline characteristics of clinical cohort.

Characteristic	Overall (N = 300)	Without advanced CKM syndrome (N = 96)	With advanced CKM syndrome (N = 204)	P-value
Age (years)	60.1± (13.4)	54.9± (13.5)	62.6± (12.4)	<0.001
Gender, n(%)				0.013
Male	194 (64.7%)	52 (54.2%)	142 (69.6%)	
Female	106 (35.3%)	44 (45.8%)	62 (30.4%)	
Smoke, n(%)				0.021
Yes	48 (16.0%)	8 (8.3%)	40 (19.6%)	
No	252 (84.0%)	88 (91.7%)	164 (80.4%)	
Hypertension, n(%)				<0.001
Yes	205 (68.3%)	46 (47.9%)	159 (77.9%)	
No	95 (31.7%)	50 (52.1%)	45 (22.1%)	
DM, n(%)				<0.001
Yes	166 (55.3%)	67 (69.8%)	99 (48.5%)	
No	134 (44.7%)	29 (30.2%)	105 (51.5%)	
HDL-C (mg/dL)	42.54 ± (11.60)	46.40 ± (11.61)	38.67 ± (7.73)	<0.001
LDL-C (mg/dL)	88.94 ± (38.67)	108.28 ± (38.67)	81.21 ± (30.94)	<0.001
TC (mg/dL)	170.15 ± (58.01)	197.22 ± (61.87)	154.68 ± (46.40)	<0.001
TG (mg/dL)	150.57 ± (203.71)	159.43 ± (292.28)	150.57 ± (141.71)	<0.001
UA (mg/dL)	5.71 ± (2.02)	4.84 ± (1.33)	6.17 ± (2.22)	<0.001
SCR (mg/dL)	1.81 ± (2.38)	0.72 ± (0.30)	2.29 ± (2.80)	<0.001
BUN (mg/dL)	39.76 ± (157.64)	19.32 ± (6.72)	47.32 ± (190.12)	<0.001
HbA1c (%)	7.6± (2.6)	8.9± (2.7)	7.1± (2.4)	<0.001
FBG (mmol/L)	6.5± (2.7)	6.7± (2.6)	6.4± (2.8)	<0.001
ALB (g/L)	37.4± (5.3)	37.2± (4.2)	36.3± (5.1)	<0.001
ALT (U/L)	21.9± (16.1)	21.8± (13.8)	21.9± (17.5)	<0.001
AST (U/L)	19.2± (14.4)	18.3± (8.9)	19.8± (16.1)	<0.001
UHR	36.1 ± (19.2)	24.8 ± (9.5)	40.6 ± (20.4)	<0.001
UAR	9.6 ± (4.1)	7.9 ± (2.7)	10.4 ± (4.5)	<0.001

We further evaluated the association and predictive performance of UHR and UAR for advanced CKM syndrome in the clinical cohort. Logistic regression analysis (**Table 5**) revealed that both UHR and UAR were significantly associated with advanced CKM syndrome across all three models (all P < 0.05). In the fully adjusted Model 3, each one-unit increase in UHR was associated with a 12% higher risk (OR = 1.12, 95% CI: 1.10-1.16, P = 0.009), while each one-unit increase in UAR was associated with a 17% higher risk (OR = 1.17, 95% CI: 1.05–1.32, P = 0.007).

**Table 5:** Association between UHR, UAR and advanced CKM syndrome in the clinical cohort.

	Model 1		Model 2		Model 3	
	OR (95% CI)	P-value	OR (95% CI)	P-value	OR (95% CI)	P-value
UHR	1.10 (1.07, 1.13)	<0.001	1.13 (1.09, 1.17)	<0.001	1.12 (1.10, 1.16)	0.009
UAR	1.24 (1.14, 1.37)	<0.001	1.27 (1.16, 1.41)	<0.001	1.17 (1.05, 1.32)	0.007

Model 1: unadjusted.

Model 2: adjusted for age, gender.

Model 3: adjusted for age, gender, smoke, hypertension, BUN, ALT, AST.

As shown in **Figure 6**, UHR demonstrated good predictive ability for advanced CKM syndrome (AUC = 0.787, 95% CI: 0.734-0.830), outperforming UAR (AUC = 0.686, 95% CI: 0.623-0.749; P < 0.001). Adding UHR to UAR did not significantly improve the AUC (combined AUC = 0.790, 95% CI: 0.737-0.843; P = 0.358 vs. UHR alone).

**Figure 6:**
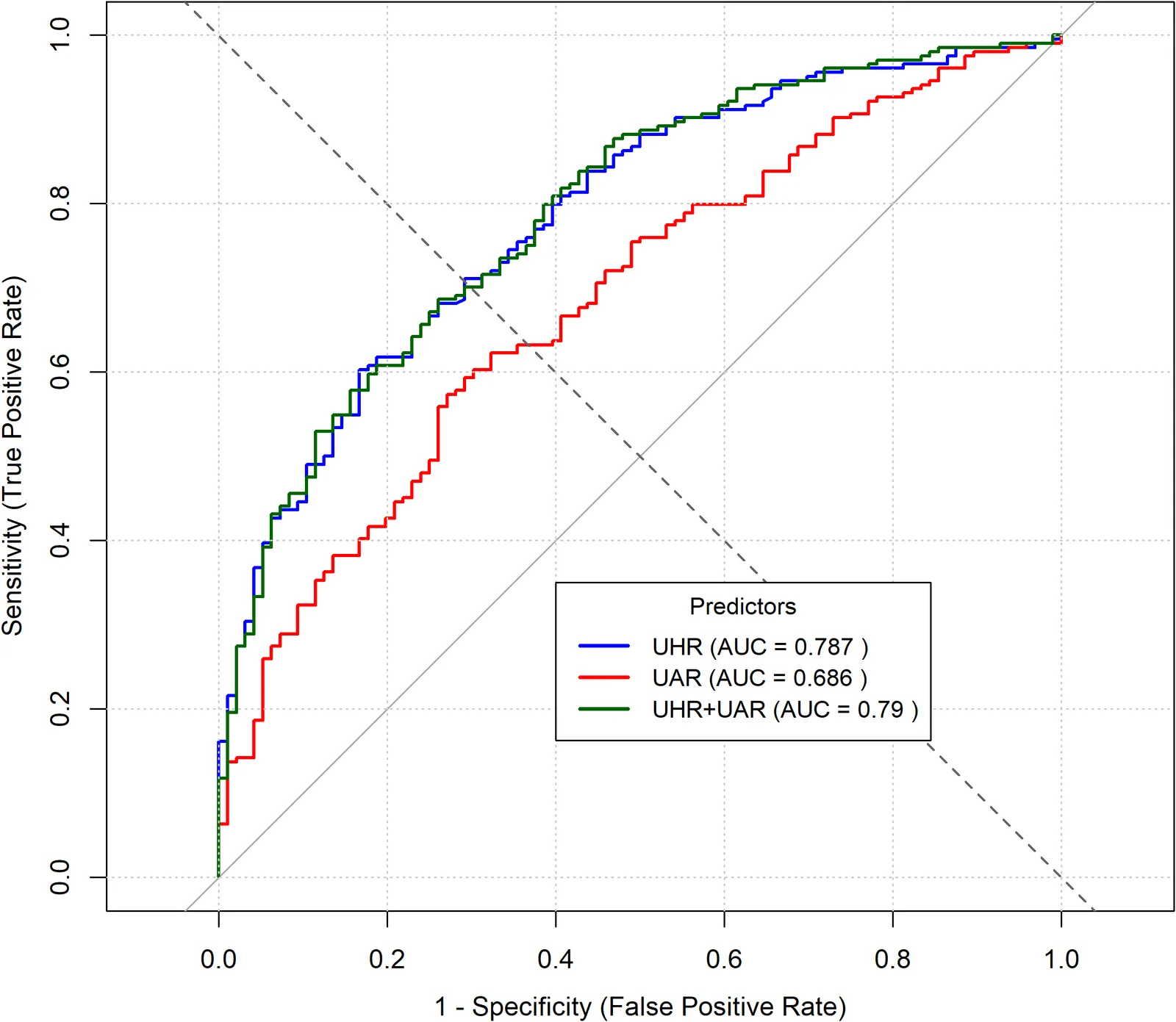
ROC curves of UHR and UAR in relation to advanced CKM syndrome in the clinical cohort.

## 4 Discussion

UHR and UAR serve as composite biomarkers that integrate metabolic, inflammatory, and nutritional information. UHR primarily captures the metabolic-inflammatory burden driven by the uric acid-HDL-C imbalance, while UAR reflects the nutritional-inflammatory status through ALB (25). Studies have demonstrated that UHR is significantly elevated in individuals with metabolic syndrome and correlates positively with insulin resistance in adolescents (26). Kolahi Ahari et al. found that UHR increased progressively with the severity of metabolic dysfunction (27). Furthermore, UHR has been shown to correlate with C-reactive protein, reinforcing its role as an inflammatory marker (27). The Dietary Inflammation Index (DII) was found to mediate 5.06% of the UHR-frailty association, highlighting the interconnections between UHR, diet, and systemic inflammation ^(^28^).^ UAR, combining UA with ALB, provides complementary information on the inflammatory-nutritional axis. As a negative acute phase reactant, ALB reflects nutritional status and possesses potent antioxidant and anti-inflammatory properties. Its decline under inflammatory conditions amplifies the deleterious effects of elevated UA (11, 12). Recent studies have demonstrated that UAR is elevated in obese adolescents and positively correlated with insulin resistance, reflecting the interplay between nutritional status and metabolic health (26). Both of these ratios can serve as convenient and low-cost indicators for reflecting the metabolic and inflammatory processes of CKM syndrome.

In this large, nationally representative cross-sectional study based on the NHANES database (2001-2020) encompassing 17,833 U.S. adults, we systematically investigated the associations of UHR and UAR with advanced CKM syndrome. It revealed that 18.21% of U.S. adults satisfied the criteria for advanced CKM syndrome. Sociodemographic factors including age, gender, race, marriage, education, and PIR were included as covariates in our multivariable models. This approach was essential to isolate the independent association of UHR and UAR with advanced CKM syndrome, given that socioeconomic disparities are well recognized drivers of cardiometabolic risk (29). Individuals with lower socioeconomic status often face higher psychosocial stress, unfavorable dietary patterns, reduced physical activity, and limited healthcare access, all of which may promote the accumulation of metabolic risk factors and accelerate CKM progression (30). By adjusting for these factors, we sought to minimize confounding and provide more robust evidence that the observed associations are specifically attributable to UHR and UAR rather than to underlying socioeconomic or demographic disparities. Results showed that elevated UHR and UAR were independently associated with an increased risk of advanced CKM syndrome across unadjusted, partially adjusted, and fully adjusted weighted logistic regression models. In addition, the RCS analysis indicated nonlinear dose-response patterns for both UHR and UAR with disease risk. Subgroup analyses further indicated that the clinical utility of UHR and UAR may differ across population strata, with UHR being more sensitive to demographic and social factors, whereas UAR may better capture biological variability related to race and socioeconomic status. ROC curve analysis demonstrated that UAR had modestly superior predictive ability for advanced CKM syndrome compared to UHR, with no additional benefit from combining the two markers. In survival analysis, both UHR and UAR remained independently associated with all-cause mortality in patients with CKM syndrome after full adjustment. Finally, these findings were externally validated in an independent clinical cohort of 300 hospitalized patients, where both UHR and UAR were significantly associated with advanced CKM syndrome, and UHR demonstrated good predictive ability, outperforming UAR.

The associations of UHR and UAR with various diseases have been increasingly recognized. Consistent with our findings, Li et al. (31) reported that UHR was an independent risk factor for decline in renal function in the hypertensive population, with a J-shaped correlation observed between UHR and eGFR slope and a cut-off point of 7.6. However, our RCS analysis identified an inflection point at UHR = 9.46, which is higher than the previously reported cut-off. This discrepancy may be attributed to differences in outcome definitions and population characteristics. Huang et al. suggested that clinicians should maintain UHR at approximately 9–10 to improve long-term prognosis in diabetic patients (32), which is similar to our identified cut-off point. Notably, for UHR, we observed a saturation effect: the risk increase was steeper below the threshold (OR = 1.153) than above it (OR = 1.057). The biological basis may lie in the interplay between UA and HDL-C. A study (33) in a hypertensive Chinese population found that the negative correlation between UA and HDL-C only existed when UA was below 7.24 mg/dL, beyond which no further association was observed. This supports the concept of a saturation threshold in the UA-HDL-C interaction captured by UHR. For UAR, we identified a threshold acceleration effect at 15.79, with a steeper risk increase above this value (OR = 1.196 vs. 1.136 below). Wang et al. (22) demonstrated a linear positive association between UAR and all-cause mortality in the general population, but our findings suggest that this relationship may become nonlinear when specifically examining CKM syndrome. The distinct nonlinear patterns of UHR and UAR suggest complementary clinical applications. UHR, with its saturation effect, may be more useful for identifying a metabolic burden threshold, beyond which further risk modification yields limited returns. UAR, with its threshold acceleration effect, may be better suited for detecting a tipping point in nutritional-inflammatory dysregulation, warranting intensive intervention when elevated.

Recent evidence has established the prognostic value of UHR and UAR across multiple clinical settings. Lei et al. reported that UHR predicted CVD events and all-cause mortality in patients with CKD (15). Consistently, our study demonstrated that UHR remained significantly associated with all-cause mortality in CKM syndrome patients, with a per-SD increase conferring a 12% higher risk in the fully adjusted model. This effect size aligns well with the prognostic value of UHR reported across different populations, underscoring its robustness as a mortality predictor. Wang et al. demonstrated that UAR predicts all-cause and cardiovascular mortality in U.S. adults from NHANES 2003–2018, with each unit increase in UAR associated with a 7% higher risk of all-cause mortality and a 9% higher risk of cardiovascular mortality (22). Our study extends these findings by demonstrating that UAR independently predicts mortality specifically in patients with CKM syndrome. Additionally, Chen et al. found that UAR was an independent risk factor for all-cause and cardiovascular mortality in patients with diabetes (34). And it can also be used to predict adverse outcomes in patients with atrial fibrillation (35). The effect size is remarkably consistent across different populations, underscoring the robustness of UAR as a prognostic marker. The combined use of these two biomarkers, by integrating metabolic, inflammatory, and nutritional dimensions, may offer a more complete evaluation of mortality risk in this population.

We found that in the ROC analysis for predicting CKM syndrome, UAR (AUC = 0.640) slightly outperformed UHR (AUC = 0.602) in the NHANES population, whereas in the clinical cohort, UHR (AUC = 0.787) substantially outperformed UAR (AUC = 0.686). This difference in predictive performance may be attributed to spectrum bias. In the community-based NHANES population, which includes relatively healthy individuals, UAR may capture subtle nutritional and inflammatory changes that precede overt metabolic dysfunction. In the hospitalized clinical cohort, where all patients have established disease, UHR-reflecting metabolic burden-becomes more discriminative. Furthermore, both biomarkers demonstrated higher predictive ability in the clinical cohort than in NHANES, likely because the NHANES cohort is population-based and includes a substantial proportion of healthy individuals with low UHR and UAR levels and limited variability, which attenuates the discriminative signal. In contrast, the clinical cohort consists entirely of hospitalized patients who are at higher risk for various diseases. These findings suggest that UHR and UAR may have different optimal application scenarios: UAR may be better suited for early risk stratification in community settings, while UHR may serve as a marker for identifying individuals with advanced metabolic derangement in clinical settings. In summary, combined with the independent association of UHR and UAR with all-cause mortality, our findings indicate that these two biomarkers offer complementary clinical value. UAR may be preferred for early screening, whereas UHR may be better suited for CKM diagnosis and metabolic risk stratification. Their combined use may facilitate prognosis evaluation and mortality risk warning.

In our study, an unexpected finding was the lower LDL-C levels in advanced CKM patients. This seemingly counterintuitive observation is consistent with the well-described “lipid paradox” in CKD and heart failure populations, where lower cholesterol levels paradoxically associate with worse outcomes, likely reflecting a state of malnutrition, chronic inflammation, and protein-energy wasting rather than a favorable metabolic profile (36). Statin use may also contribute (37). Moreover, qualitative lipoprotein abnormalities, such as increased small dense LDL particles, may persist even when LDL-C concentration is normal, and this phenotype is considered a more potent atherogenic stimulus than elevated LDL-C alone (38). In our clinical cohort, despite lower LDL-C, the TG/HDL-C ratio remained significantly elevated, supporting this explanation (39). Thus, lipid assessment in CKM syndrome should consider both quantitative and qualitative measures.

This study has several important strengths. First, to our knowledge, this is the first study to comprehensively evaluate and directly compare UHR and UAR for predicting CKM syndrome using a large, nationally representative sample with 20 years of data. Second, we employed rigorous statistical methods, including weighted multivariable analyses accounting for the complex NHANES sampling design, RCS analysis to explore nonlinear relationships, and comprehensive subgroup analyses. Third, we validated our findings in an independent clinical cohort, which substantially strengthens the robustness and generalizability of our conclusions. Fourth, we examined both cross-sectional (CKM syndrome diagnosis) and longitudinal (all-cause mortality) outcomes. Nevertheless, several limitations should be acknowledged. First, the cross-sectional design of the NHANES study precludes causal inference. Second, data on medication use that could influence UA, ALB and HDL-C levels were not fully accounted for. In the NHANES cohort, missing data for these variables were substantial, and including them would have compromised statistical power; in the clinical cohort, reliable preadmission medication histories were not consistently documented. Third, the clinical validation cohort was derived from a single tertiary hospital in China, which may limit generalizability. Fourth, the relatively modest AUCs in the NHANES population suggest that neither UHR nor UAR alone is sufficient as a standalone screening tool in the general population. Fifth, the single-timepoint measurement of UA, HDL-C and ALB may not reflect long-term exposure or within individual variability over time. Hence, future prospective studies with comprehensive medication records and longitudinal follow-up are needed to confirm and extend these findings.

## 5 Conclusion

Our results provide compelling evidence of a significant positive association between UHR, UAR and advanced CKM syndrome. Both of the two biomarkers remained independently associated with all-cause mortality in CKM patients after full adjustment. These findings suggest that UHR and UAR are simple, cost-effective biomarkers that may facilitate risk stratification for CKM syndrome, with potential complementary applications: UAR may be preferred for early screening, whereas UHR may be better suited for CKM diagnosis and metabolic risk stratification. Their combined use may facilitate prognosis evaluation and mortality risk warning.

## Data Availability

The datasets presented in this study can be found in online repositories. The names of the repository/repositories and accession number(s) can be found below: https://www.cdc.gov/nchs/nhanes/.

## References

[1] Ndumele CE Rangaswami J Chow SL Neeland IJ Tuttle KR Khan SS . Cardiovascular-kidney-metabolic health: A presidential advisory from the American Heart Association. Circulation. (2023) 148:1606–35. doi: 10.1161/cir.0000000000001184 37807924

[2] Aggarwal R Ostrominski JW Vaduganathan M . Prevalence of cardiovascular-kidney-metabolic syndrome stages in US adults, 2011-2020. JAMA. (2024) 331:1858–60. doi: 10.1001/jama.2024.6892 38717747 PMC11079779

[3] Wen S Arakawa H Tamai I . Uric acid in health and disease: From physiological functions to pathogenic mechanisms. Pharmacol Ther. (2024) 256:108615. doi: 10.1016/j.pharmthera.2024.108615 38382882

[4] Saito Y Tanaka A Node K Kobayashi Y . Uric acid and cardiovascular disease: A clinical review. J Cardiol. (2021) 78:51–7. doi: 10.1016/j.jjcc.2020.12.013 33388217

[5] Sellmayr M Hernandez Petzsche MR Ma Q Krüger N Liapis H Brink A . Only hyperuricemia with crystalluria, but not asymptomatic hyperuricemia, drives progression of chronic kidney disease. J Am Soc Nephrol. (2020) 31:2773–92. doi: 10.1681/asn.2020040523 32938648 PMC7790211

[6] Van Linthout S Frias M Singh N De Geest B . Therapeutic potential of HDL in cardioprotection and tissue repair. Handb Exp Pharmacol. (2015) 224:527–65. doi: 10.1007/978-3-319-09665-0_17 25523001

[7] Rader DJ Hovingh GK . HDL and cardiovascular disease. Lancet. (2014) 384:618–25. doi: 10.1016/s0140-6736(14)61217-4 25131981

[8] von Eckardstein A Nordestgaard BG Remaley AT Catapano AL . High-density lipoprotein revisited: Biological functions and clinical relevance. Eur Heart J. (2023) 44:1394–407. doi: 10.1093/eurheartj/ehac605 36337032 PMC10119031

[9] Aktas G . An overview of the role of serum uric acid to high-density lipoprotein cholesterol ratio in type 2 diabetes mellitus and in other inflammatory and metabolic conditions. Minerva Med. (2025) 116:405–15. doi: 10.23736/s0026-4806.25.09712-5 40598948

[10] Yin J Zheng C Li Z Chang Y Cao L Qu Y . Gender differences in the association between the uric acid to high-density lipoprotein cholesterol ratio and diabetes risk: A mediation analysis of c-reactive protein, triglycerides, and insulin resistance. Lipids Health Dis. (2024) 23:409. doi: 10.1186/s12944-024-02404-6 39696515 PMC11654205

[11] Belinskaia DA Voronina PA Goncharov NV . Integrative role of albumin: Evolutionary, biochemical and pathophysiological aspects. J Evol Biochem Physiol. (2021) 57:1419–48. doi: 10.1134/s002209302106020x 34955553 PMC8685822

[12] Belinskaia DA Voronina PA Shmurak VI Jenkins RO Goncharov NV . Serum albumin in health and disease: Esterase, antioxidant, transporting and signaling properties. Int J Mol Sci. (2021) 22:10318. doi: 10.3390/ijms221910318 34638659 PMC8508759

[13] Qin A Yang D Wang S Dong L Tan J Tang Y . Uric acid-based ratios for predicting renal failure in Chinese IgA nephropathy patients. Int J Med Sci. (2023) 20:1584–91. doi: 10.7150/ijms.85430 37859692 PMC10583186

[14] Fan Z Feng C Li X Zhang Z Gong H Di J . Association between uric acid to high-density lipoprotein cholesterol ratio and chronic kidney disease in middle-aged and older adults: Results from two nationally representative population-based study. Ren Fail. (2025) 47:2575920. doi: 10.1080/0886022x.2025.2575920 41146462 PMC12570220

[15] Lei K Hu S Zhang Y Wang J Wang J . Uric acid to high-density lipoprotein cholesterol ratio in association with adverse health outcomes in patients with chronic kidney disease. Nutr Metab Cardiovasc Dis. (2025) 35:104161. doi: 10.1016/j.numecd.2025.104161 40450421

[16] Yi Y Luo Q Chen J Chen Z Aydemir HA Chen P . Association between the uric acid-to-HDL-cholesterol ratio (UHR) and the risk of cardiovascular disease and dyslipidemia: A population-based study. Lipids Health Dis. (2025) 24:143. doi: 10.1186/s12944-025-02551-4 40241174 PMC12001538

[17] Sun S Xu H Liu L Luan Z Liu C Zhi F . Association of serum uric acid-to-high-density lipoprotein cholesterol ratio (UHR) with risk of myocardial infarction among individuals with diabetes: A cross-sectional analysis using data from NHANES 2005-2020. Eur J Med Res. (2025) 30:554. doi: 10.1186/s40001-025-02845-4 40605038 PMC12219884

[18] Yan C Zhen Z Shi Y Zhao P Song X Peng Q . Association between uric acid to high-density lipoprotein cholesterol ratio and peripheral arterial disease in hypertensive patients: A cohort study. Nutr Metab Cardiovasc Dis. (2025) 35:104026. doi: 10.1016/j.numecd.2025.104026 40210499

[19] Yalcinkaya D Karacali K Ilhan BC Yarlioglues M . Relation between serum uric acid to albumin ratio and severity of chronic coronary artery disease. (2024) 75:386–93. doi: 10.1177/00033197231161902 36912476

[20] Şaylık F Çınar T Selçuk M Tanboğa İH . The relationship between uric acid/albumin ratio and carotid intima-media thickness in patients with hypertension. Arq Bras Cardiol. (2023) 120:e20220819. doi: 10.36660/abc.2022081910.36660/abc.20220819 PMC10124582 37098960

[21] Oflar E Yıldız C Koyuncu A Mavi B Karabulut D Çağlar FNT . Relationship between uric acid albumin ratio and peripheral artery disease complexity. Eur Rev Med Pharmacol Sci. (2023) 27:11472–8. doi: 10.26355/eurrev_202312_34586 38095395

[22] Wang G Li G Wang P Zang M Pu J . The uric acid to albumin ratio predicts all-cause and cardiovascular mortality among U.S. adults results from the National Health and Nutrition Examination Survey in 2003-2018. Int J Med Sci. (2025) 22:2277–88. doi: 10.7150/ijms.106664 40386059 PMC12080566

[23] Zhang T Man ZY Zhang SM Mu JJ Liao YY . A metabolic-to-inflammatory pattern in cardiovascular-kidney-metabolic syndrome staging: A comparative cross-sectional study. Front Endocrinol (Lausanne). (2026) 17:1805355. doi: 10.3389/fendo.2026.1805355 42100200 PMC13143627

[24] Kim HJ Kim BS Kim H Lee J Jo HH Kim DW . Long-term clinical outcome and risk stratification across stages of cardiovascular-kidney-metabolic syndrome in a nationwide cohort. Korean J Intern Med. (2025) 40:975–89. doi: 10.3904/kjim.2025.194 41146593 PMC12611481

[25] Chen H Qiu M Zhang H Huai Y Huang Q Li P . Uric acid and HDL-C-derived inflammatory ratios as biomarkers in schizophrenia and major depressive disorder: A retrospective study. Front Psychiatry. (2025) 16:1698455. doi: 10.3389/fpsyt.2025.1698455 41256944 PMC12620466

[26] Dikker O Aktaş A Şahin M Doğan M Dağ H . The association of serum uric acid levels and various uric acid-related ratios with insulin resistance and obesity: A preliminary study in adolescents. Children (Basel). (2023) 10:1493. doi: 10.3390/children10091493 37761454 PMC10528166

[27] Kolahi Ahari R Mansoori A Sahranavard T Miri MS Feizi S Esmaily H . Serum uric acid to high-density lipoprotein ratio as a novel indicator of inflammation is correlated with the presence and severity of metabolic syndrome: A large-scale study. Endocrinol Diabetes Metab. (2023) 6:e446. doi: 10.1002/edm2.446 37605374 PMC10638626

[28] Huang Y Wang X Yin X Li Z . The ratio of serum uric acid to high-density lipoprotein cholesterol as a predictor for frailty prevalence and mortality in the elderly U.S. adults. Lipids Health Dis. (2025) 24:325. doi: 10.1186/s12944-025-02756-7 41094549 PMC12522384

[29] Zhu R Wang R He J Wang L Chen H Niu X . Prevalence of cardiovascular-kidney-metabolic syndrome stages by social determinants of health. JAMA Netw Open. (2024) 7:e2445309. doi: 10.1001/jamanetworkopen.2024.45309 39556396 PMC11574692

[30] Li J Lei L Wang W Ding W Yu Y Pu B . Social risk profile and cardiovascular-kidney-metabolic syndrome in US adults. J Am Heart Assoc. (2024) 13:e034996. doi: 10.1161/jaha.124.034996 39136302 PMC11963957

[31] Li S Liu Z Lu C . Association of uric acid to high-density lipoprotein cholesterol ratio with the presence or absence of hypertensive kidney function: Results from the China Health and Retirement Longitudinal Study (CHARLS). BMC Nephrol. (2025) 26:123. doi: 10.1186/s12882-024-03939-7 40050799 PMC11884189

[32] Huang X Hu L Li J Wang X . U-shaped association of uric acid to HDL cholesterol ratio (UHR) with ALL-cause and cardiovascular mortality in diabetic patients: NHANES 1999-2018. BMC Cardiovasc Disord. (2024) 24:744. doi: 10.1186/s12872-024-04436-3 39725874 PMC11674183

[33] Yu Y Huang X Li M Ding C Hu L Zhong X . Is there a nonlinear relationship between serum uric acid and lipids in a hypertensive population with eGFR ≥30 ml/min/1.73 m(2)? Findings from the China Hypertension Registry Study. Int J Endocrinol. (2020) 2020:9725979. doi: 10.1155/2020/9725979 33014048 PMC7519986

[34] Chen S Zhang M Hu S Shao X Liu L Yang Z . Uric acid to albumin ratio is a novel predictive marker for all-cause and cardiovascular death in diabetic patients: A prospective cohort study. Front Endocrinol (Lausanne). (2024) 15:1388731. doi: 10.3389/fendo.2024.1388731 39911231 PMC11794066

[35] Gong A Cao Y Li Z Li F Li W Ren B . Uric acid-to-albumin ratio as a cardiometabolic marker for predicting adverse outcomes in patients with atrial fibrillation: Evidence from two independent cohorts. Front Endocrinol (Lausanne). (2026) 17:1786997. doi: 10.3389/fendo.2026.1786997 41767383 PMC12945761

[36] Liu Y Coresh J Eustace JA Longenecker JC Jaar B Fink NE . Association between cholesterol level and mortality in dialysis patients: Role of inflammation and malnutrition. Jama. (2004) 291:451–9. doi: 10.1016/j.accreview.2004.03.075 14747502

[37] Collins R Reith C Emberson J Armitage J Baigent C Blackwell L . Interpretation of the evidence for the efficacy and safety of statin therapy. Lancet. (2016) 388:2532–61. doi: 10.1016/s0140-6736(16)31357-5 27616593

[38] Hoogeveen RC Gaubatz JW Sun W Dodge RC Crosby JR Jiang J . Small dense low-density lipoprotein-cholesterol concentrations predict risk for coronary heart disease: The Atherosclerosis Risk In Communities (ARIC) study. Arterioscler Thromb Vasc Biol. (2014) 34:1069–77. doi: 10.1161/atvbaha.114.303284 24558110 PMC3999643

[39] Moriyama K . The association between the triglyceride to high-density lipoprotein cholesterol ratio and low-density lipoprotein subclasses. Intern Med. (2020) 59:2661–9. doi: 10.2169/internalmedicine.4954-20 32669498 PMC7691041

