# Supplementary material for: Uric acid-based nutritional and inflammatory biomarkers for predicting cardiovascular-kidney-metabolic syndrome and mortality: a population-based study with external validation
Source: Front Endocrinol (Lausanne). 2026 Sep 22;17:1937815. doi: 10.3389/fendo.2026.1937815 (PMC13638566; doi:10.3389/fendo.2026.1937815)
Supplement: Supplementary file 1 [file Table1.docx]

**Table S1. Definition of CKM syndrome stages**

| **CKM Stages** | **Conditions** |
| --- | --- |
| **Stage 0** | Individuals without any risk factors for CKM syndrome:  a. Normal body mass index (BMI < 23kg/m^2^ for Asian; < 25kg/m^2^ for others)  b. Normal waist circumference (WC < 80 cm for Asian women; <90 cm for Asian men; <88 cm for other women; <102 cm for other men)  c. Normal fasting blood glucose (FBG < 100 mg/dL) and glycated hemoglobin (HbA1c < 5.7%)  d. Normal blood pressure (SBP < 130 mmHg and DBP < 80 mmHg)  e. Normal lipid profile (TG < 150 mg/dL)  f. No evidence of chronic kidney disease (CKD) or subclinical/clinical cardiovascular disease (CVD) |
| **Stage 1** | Individuals without the presence of other metabolic risk factors or CKD but with any of the following:  a. Elevated BMI ( ≥ 23 kg/m² for Asians; ≥ 25 kg/m² for others)  b. Elevated WC ( ≥ 80 cm for Asian women; ≥ 90 cm for Asian men; ≥ 88 cm for other women; ≥ 102 cm for other men)  c. Prediabetes (FBG ≥ 100-124 mg/dL or HbA1c between 5.7% and 6.4%) |
| **Stage 2** | Individuals with moderate-to-high-risk CKD per KDIGO guidelines or metabolic risk factors as follows:   a. Elevated fasting TG ( ≥ 135 mg/dL)   b. Hypertension (SBP ≥ 130 mmHg, DBP ≥ 80 mmHg, or use of antihypertensive medications)  c. Diabetes (HbA1c ≥ 6.5%, use of glucose-lowering medication, or self-reported physician diagnosis)  d. Metabolic syndrome ( ≥ 3 of the following 5 conditions: elevated WC (≥ 88 cm in women or ≥ 102 cm in men), low HDL-C [ < 40 mg/dL for men, < 50 mg/dL for women], fasting TG ≥ 150 mg/dL, elevated blood pressure [SBP ≥ 130 mmHg, DBP ≥ 80 mmHg, or antihypertensive use], or prediabetes). |
| **Stage 3** | Individuals with any of the following:  a. Very-high-risk CKD (stage G4 or G5 CKD or very high risk per KDIGO classification determined by GFR and urinary albumin-to-creatinine ratio)  b. High cardiovascular risk (10-year CVD risk ≥ 20%, calculated using AHA PREVENT equations based on recommended thresholds [https://professional.heart.org/en/guidelines-and-statements/prevent-calculator]) |
| **Stage 4** | Individuals with self-reported history of coronary heart disease, angina pectoris, congestive heart failure, myocardial infarction, or stroke.  Stage 4a: without kidney failure; estimated glomerular filtration rate ≥ 15 ml/min/1.73m².  Stage 4b: with kidney failure; estimated glomerular filtration rate < 15 ml/min/1.73m². |
